# 

**Published:** 1836-01

**Authors:** 


### CONTENTS OF No. I.

OF THE

### BRITISH AND FOREIGN MEDICAL REVIEW.

### Part First.—Analytical and Critical Reviews.

PAGE

Art. I.—Francisci Bene, Med. Doct., Consiliarii Regii, Professoris P. O. Therapiæ Specialis ac Praxis Medicæ, et Senioris Facultatis Medicæ in Regia Scientiarum Universitate Hungarica, Elementa Medicinæ Practicæ. E Prælectionibus illius publicis Edita per Franciscum Bene, Jun. m.d. . 1

Art. II.—Memoir of the Life and Medical Opinions of John Armstrong, m.d. formerly Physician to the Fever Institution of London, &c. To which is added, an Inquiry into the Facts connected with those Forms of Fever attributed to Malaria or Marsh Effluvium. By Francis Boott, m.d. &c. . . 34

Lectures on the Morbid Anatomy, Nature, and Treatment of Acute and Chronic Diseases, delivered in the Theatre of Anatomy, Webb street, by the late John Armstrong, m.d. &c. Edited by Joseph Rix, m.r.c.s. . . ib.

Art. III.—A Treatise on Pulmonary Consumption; comprehending an Inquiry into the Causes, Nature, Prevention, and Treatment of Tuberculous and Scrofulous Diseases in general. By James Clark, m.d. f.r.s. . . 70

Art. IV.—Die geburtshülfliche Exploration, von Dr. Anton Friedrich Hohl, ausserordentlichem Professor der Universität zu Halle, u. s. w. Erster Theil: Das Hören.—Zweiter Theil: Das explorative Sehen und Fühlen, nebst einem Anhange . . . . . . . . 85

Operationslehre für Geburtshelfer, in zwei Theilen. Von Dr. Hermann Friedrich Kilian, ordentl. öffentl. Professor der Geburtshülfe und der Geburtshülflichen Klinik an der Rheinischen Friedrichs-Wilhelms Universität, u. s. w. Erster Theil: die Operative Geburtshülfe . . . ib.

Art. V.—Illustrations of the Elementary Forms of Disea [illegible]. By Robert Carswell, m.d., Professor of Pathological Anatomy in the University of London. Eight Fasciculi . . . . . 117

Art. VI.—The American Cyclopædia of Practical Medicine and Surgery; a Digest of Medical Literature. Edited by Isaac Hays, m.d. Parts I. to VII. . 152

### Part Second.—Bibliographical Notices.

Art. I.—Pathological Researches on Phthisis. By E. Ch. Louis, m.d., Physician to the Hospital of La Pitié, &c. Translated from the French, with Introduction, Notes, Additions, and an Essay on Treatment, by Charles Cowan, m.d. 165

Art. II.—An Exposition of the Nature, Treatment, and Prevention of Continued Fever. By Henry M'Cormac, m.d. . . . . 172

Art. III.—The Cyclopædia of Practical Medicine. Edited by John Forbes, m.d. f.r.s., Alexander Tweedie, m.d., and John Conolly, m.d. . 176

Art. IV.—Von der blutigen Kopfgeschwulst der Neugeborenen. By Karl Unger, Professor in the Königsberg University, &c. Beiträge zur Klinik der Chirurgie . 182

4218

PAGE

Observations on Sanguineous Tumours of the Head which form spontaneously, sometimes denominated Cephalæmatoma, and Abscessus Capitis Sanguineus Neonatorum. By E. GEDDINGS, M.D. . . . . 182

ART. V.—The Clinique Médicale; or Reports of Medical Cases. By G. ANDRAL. Condensed and translated, with Observations extracted from the Writings of the most distinguished Medical Authors, by D. SPILLAN, M.D. Part I.-II.-III. . 188

ART. VI.—Natur und Kunst in heilung der Krankheiten: ein Leitfaden für angehende Aerzte. Von Dr. JOHANN JACOB GÜNTHER . . 191

ART. VII.—A Therapeutic Arrangement and Syllabus of Materia Medica. By JAMES JOHNSTONE, M.D. . . . . . 193

ART. VIII.—De l'Influence des Professions sur la Durée de la Vie. Recherches Statistiques. Par le Dr. H. C. LOMBARD . . . . 194

ART. IX.—Introductory Lecture to a Course of Lectures on the Practice of Physic, to be delivered at the Medical School in Aldersgate street. By MARSHALL HALL, M.D. F.R.S.L. and E., &c. . . . . 203

Syllabus of Lectures on the Diseases of the Nervous System; to be delivered in the Aldersgate School of Medicine. By MARSHALL HALL, M.D., &c. . ib.

An Introductory Address, delivered at the commencement of the Tenth Session of the Manchester School of Medicine and Surgery, Pine street. By JAMES L. BARDSLEY, M.D., &c. . . . . . ib.

An Address delivered at the First Anniversary Meeting of the Birmingham School of Medicine and Surgery. By JAMES THOMAS LAW, Chancellor of the Diocess of Litchfield . . . . . . ib.

An Introduction to the Study of Practical Medicine; being an Outline of the leading Facts and Principles of the Science, as taught in a Course of Lectures, delivered in the Marischal College of Aberdeen. By JOHN MACROBIN, M.D. ib.

St. Thomas's Hospital Reports. No. I. By JOHN F. SOUTH, Assistant Surgeon ib.

ART. X.—A Memoir of JAMES JACKSON, Jun. M.D.; with Extracts from his Letters to his Father, and Medical Cases collected by him. By JAMES JACKSON, M.D., Professor of the Theory and Practice of Physic in Harvard University; and Physician of the Massachusetts General Hospital . . 209

ART. XI.—Compendium of the Ligaments; illustrated by Woodcuts, with the Articular Cartilages, Interarticular or Moveable Fibro-Cartilages, Synovial Membranes, and Bursæ Mucosæ of the Joints, &c. By A. M'NAB, Jun. M.R.C.S. 215

ART. XII.—Illustrations of the Botany and other Branches of the Natural History of the Himalayan Mountains, and of the Flora of Cashmere. By J. FORBES ROYLE, Esq., F.L.S. &c. . . . . . ib.

ART. XIII.—The British Medical Almanack, and Supplement . . 216

ART. XIV.— THE FOREIGN JOURNALS.

THE GERMAN JOURNALS: . . . . . 217
1. Journal der practischen Heilkunde . . . . 218
2. Die Bibliothek der practischen Heilkunde . . . ib.
3. Journal der Chirurgie und Augen-Heilkunde . . . ib.
4. Neue wissentschaftliche Annalen der gesammten Heilkunde . 219
5. Medicinische Jahrbücher des kaiserl. königl. Oesterreichischen Staates ib.
6. Allgemeines Repertorium der gesammten deutschen Medizinisch-chirurgischen Journalistik . . . . 220

ITALIAN JOURNALS: . . . . . . 221
1. Antologia Medica . . . . . 222
2. Annali Universali di Medicina . . . . ib.
3. Giornale delle Scienze Medico-Chirurgiche . . . 223

PAGE

French Journals: . . . . . 223

1. Revue Médicale, Française, et étrangère, et Journal de Chirurgie, &c. 225

2. Archives de Médecine, Journal complémentaire des Sciences Médicales ib.

3. Gazette Médicale de Paris . . . . ib.

4. La Lancette Française; Gazette des Hôpitaux . . . 226

5. Annales d'Hygiène et de Médecine Légale . . . ib.

Danish Journals: . . . . . . 227

1. Bibliothek for Læger . . . . ib.

2. Journal for Medicin og Chirurgie . . . . 228

8. Eyr, et Medicinsk Tidsskrift . . . . ib.

American Journals: . . . . . ib.

1. The American Journal of the Medical Sciences . . . 229

2. The North American Archives of Medical and Surgical Science . 230

### Part Third.—Selections from Foreign Journals.

### ANATOMY.

On the Structure of Cartilage and Bone in the Human Subject. By W. and F. Arnold. (*With Engravings*, Plate I.) . . . . 231

On the Modifications of Structure in the Thorax and Lungs, produced by Age. By MM. Hourmann et Dechambre. (*With Engravings*, Plate II.) . 233

On the Intimate Structure of the Intestinal Glands. By Dr. Boehm . . 236

### MORBID ANATOMY.

On the Anatomical Characters of Cholera. By W. E. Horner, m.d. . ib.

On Tubercles. By J. A. Rochoux, Physician to l'Hospice de la Vieillesse (Hommes) 238

### PHYSIOLOGY.

On the Early Development of the Human Embryo. By Prof. von Baer. (*With Engravings*, Plate II.) . . . . . . 239

The Pulmonary Exhalation experimentally investigated. By Prof. Tiedemann . 241

Temperature of the Body in Disease. By MM. Becquerel and Breschet . 246

### MEDICINE,

PATHOLOGICAL, PRACTICAL, AND THERAPEUTICAL.

On the Plague now prevalent in Egypt. By Clot-Bey . . . 247

On the Effects of Nux Vomica and Aconite on Animals. By Dr. Lombard . 248

Clinical Report of the Academical Years 1832-33 and 33-34. By G. Del Chiappa, Professor of Clinical Medicine, Pavia . . . 249

On the Pneumonia of newly-born Children. By Dr. Kluge, of Berlin . 253

Pathology of Rheumatism. By M. R. Parise . . . 254

On the Employment of Nux Vomica and its Preparations in the Treatment of Dysentery. By E. Geddings, m.d. . . . . . 255

On the Use of Purified Alumina in Infantile Cholera, and of Sulphate of Copper in Softening of the Stomach. By Dr. G. E. F. Dürr . . . 257

On Hæmorrhagic Pericarditis. By Dr. Seidlitz, Senior Physician to the Naval Hospital at St. Petersburg . . . . . . 259

On the Use of the Distilled Water of the Prunus Lauro-Cerasus in Facial Neuralgia. By E. S. Bennett, m.d. . . . . 263

PAGE

Clinical Observations on the Effects of certain Medicines on the Heart. By Dr. LOMBARD, of Geneva . . . . . . 264

On the Action of Creosote: Report of M. SOLON to the Academy of Medicine . 265

Experiments and Observations of the Powers of Creosote on Man and Animals. By Dr. GUISEPPE CORNELIANI, of Pavia . . . . ib.

SURGERY.

Observations on Staphyloraphy, or Palate-Suture, and on Extirpation of the Tonsils. By N. R. SMITH, M.D., of Maryland. (*With Woodcuts.*) . ib.

On the Cure of Fistulæ and Ulcers. By Dr. GOTTLIEB CRAMER . . 268

On permanent Retraction of the Fingers. By M. GOYRAUD, D.M. . . 269

On the Treatment of Ozœna and other Diseases of the Organ of Smell. By Dr. JAMES HERON, of New York . . . . . 270

New Treatment of Blenorrhagia in Women. By P. RICORD, Surgeon, of Paris 271

MEDICAL STATISTICS.

Historical and Statistical Account of la Maison Royale de Charenton. By M. ESQUIROL . . . . . . . 272

Official Report of the State of Lunatics in Norway, in the year 1825. By FREDERICK HOLST, M.D. . . . . . 278

On the Means of increasing the Number of Leeches in France. By M. GUIBOURT 281

HYGIENE.

On the Diseases of Printers. By A. CHEVALLIER . . . 282

MENTAL DISORDERS.

Moral Management of the Insane: PINEL unchaining the Lunatics . . 286

CHEMISTRY.

Comparative Analysis of White and Grey Cerebral Matter. By M. JOHN . 288

Solidification of Carbonic Acid Gas. By M. ARAGO . . . ib.

---

### PART FOURTH.—Medical Intelligence.

ON THE PRESENT STATE OF MEDICINE IN PRUSSIA, by J. F. C. HECKER, M.D., Professor of Medicine in the University of Berlin.—*First Report:* Of the Medical Institutions and the Medical Profession . . . 289

On the present State of Medicine in Spain . . . . 303

Public Health.—Mr. BUCKINGHAM'S Bill for establishing Public Walks, Playgrounds, Baths, &c. . . . . . 304

The King of Sweden and M. GAMA . . . . . 305

Responsibility of Practitioners—Poisoning by mistake . . . 306

MEDICAL CORPORATIONS . . . . . ib.

Study of Materia Medica . . . . . . 307

Dinner to Dr. BALMANNO, of Glasgow . . . . ib.

Lunatic Asylum on an improved Plan: The Midland Retreat . . 308

OBITUARY . . . . . . . ib.

LIST of ORIGINAL PAPERS in the British Journals during the last Quarter . 309

LIST of BOOKS received for Review . . . . . 311

